# May-Thurner Syndrome With Pulmonary Embolism as the First Presentation Rather Than Deep Vein Thrombosis

**DOI:** 10.7759/cureus.509

**Published:** 2016-02-24

**Authors:** Adebayo A Fasanya, Gina LaCapra

**Affiliations:** 1 Pulmonary and Critical Care Medicine, Allegheny General Hospital; 2 Internal Medicine, Overlook Medical Center

**Keywords:** pulmonary embolism, femoral, dvt, iliofemoral, young female, venogram, anticoagulation

## Abstract

May-Thurner syndrome (MTS) is a rare disease that causes deep vein thrombosis (DVT) in young females (age 20 to 50). DVT is caused by mechanical obstruction of the left common iliac vein by the right common iliac artery resulting in stasis rather than a primary hypercoagulable state. Although MTS is found in 22% of cadavers, it causes <5% of lower extremity venous disorder. Greater than 70% compression is needed to cause DVT. MTS patients usually present with acute left leg edema. Many cases are recurrent with a past workup negative for other etiologies of DVT or pulmonary embolism (PE). Cases rarely present as PE rather than DVT. We present a case of this syndrome at a younger-than-typical age with PE as the first presentation. Femoral stick venogram is the gold standard for diagnosing MTS as therapeutic procedures can be done concurrently. Anticoagulation therapy alone is insufficient to prevent recurrence.

## Introduction

May-Thurner syndrome (MTS) is a rare condition characterized by the compression of the left common iliac vein by the overlying right common iliac artery against the underlying vertebral body [1]. This leads to the stasis of blood, predisposing the patient to blood clots. MTS is most commonly seen in women between the ages of 20 and 50 [1]. Although this condition is present in up to 22% of cadavers, it is rarely diagnosed [2]. This case report discusses a 19-year-old female who presented with shortness of breath and chest pain for several days and was found to have a submassive pulmonary embolism (PE). Further workup did not indicate a hypercoagulable state and venography revealed MTS. Although she did not report leg pain or edema, the lower extremity doppler showed common femoral, great saphenous, profunda, popliteal, posterior tibial, and peroneal veins thrombosis. Interventions included anticoagulation, thrombolysis, stenting of the left iliac vein, and placement of an inferior vena cava (IVC) filter. Anticoagulation therapy alone is not enough to prevent recurrence [3]. It is important to consider this syndrome as part of the differential diagnosis in a young patient who presents with PE without obvious etiology.

## Case presentation

**Figure 1:**
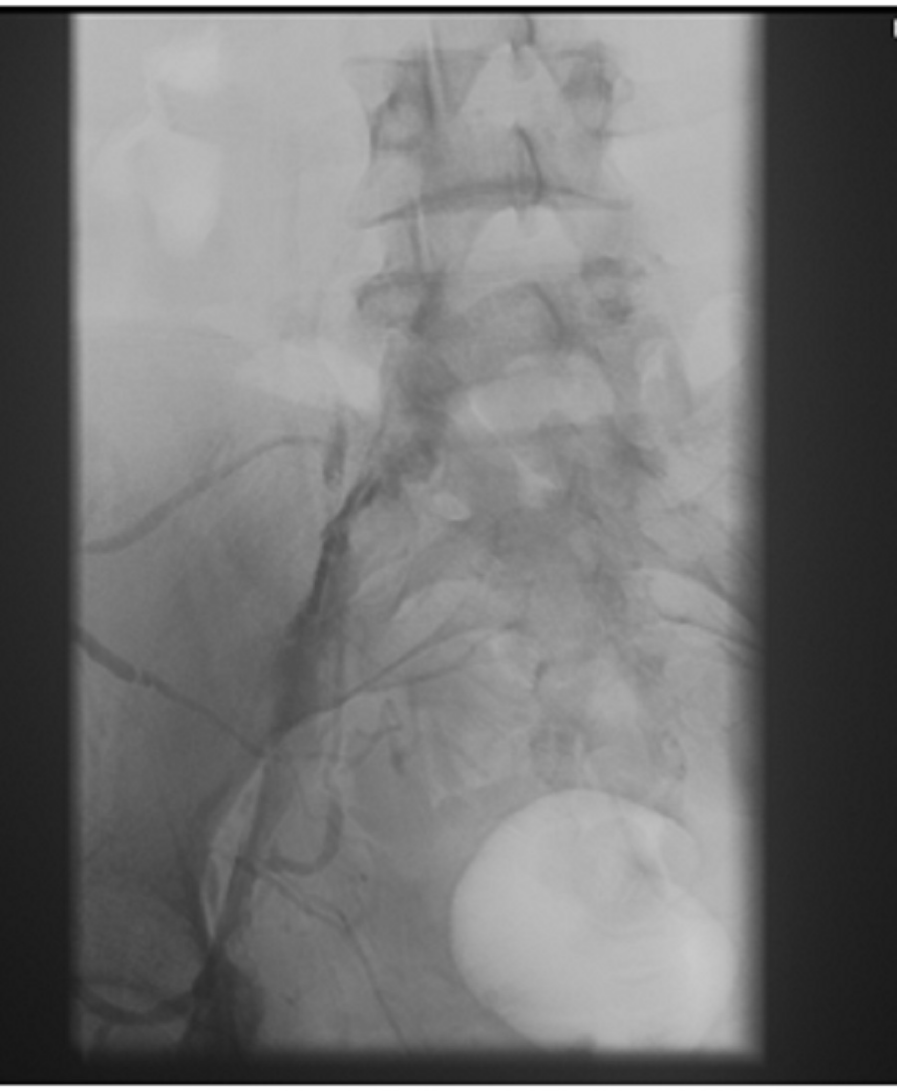
Difference in iliac vein caliber and presence of collateral before stent deployment.

**Figure 2:**
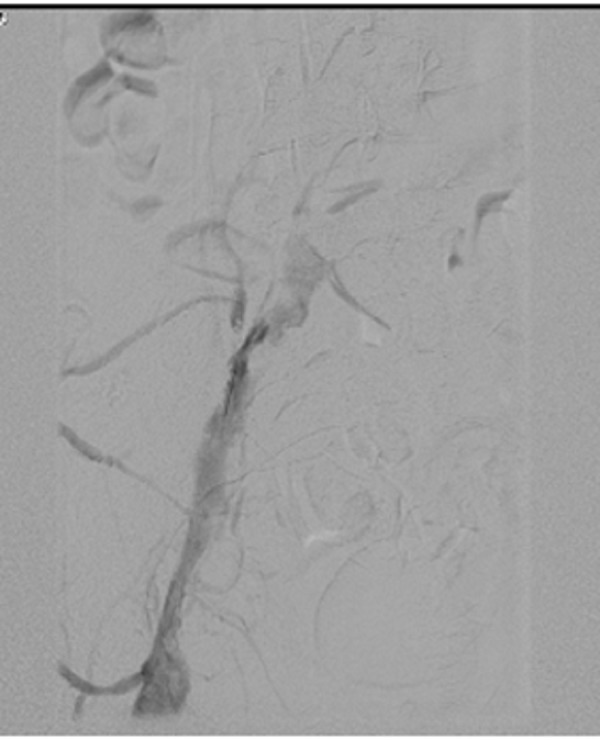
Difference in iliac vein caliber and presence of collateral before stent deployment.

We present the case of a 19-year-old white female with a past medical history significant for anxiety and depression, who was in her usual state of health until two days before presentation when she developed shortness of breath and pleuritic pain under her right breast. She denied recent leg edema or pain. She has no history of thromboembolic disease; however, her father had deep vein thrombosis (DVT) and PE following knee surgery. She denied tobacco, alcohol, or illicit drug use. She is obese (BMI 42) and tachycardic but normotensive. Her lungs were clear to auscultation bilaterally, her chest X-ray was clear, and her electrocardiogram revealed sinus tachycardia. A computed tomography (CT) scan of her chest with contrast revealed bilateral pulmonary emboli beginning at the level of the lobar vessels extending into the segmental and subsegmental branches. She was admitted to the intensive care unit where she was started on intravenous heparin infusion. Lower extremity dopplers revealed acute DVT in the left common femoral, greater saphenous, femoral profunda, popliteal, posterior tibial, and peroneal veins. Transthoracic echocardiogram showed hypokinesis of the right ventricular free wall with sparing of the apical segment, which constitutes a McConnell sign. This is suggestive of pulmonary embolism with right heart strain. The vascular surgery team evaluated her, and due to her extensive clot burden, she underwent catheter-directed thrombolysis of the pulmonary artery. Pressure measurements showed marked improvement in the pulmonary artery pressures (preprocedure: 59/34, postprocedure: 41/24). Her hospital course was complicated by a drop in her platelet count and a positive heparin-induced thrombocytopenia antibody. The patient was switched from heparin to argatroban. Her hypercoagulability workup, which included lupus anticoagulant, anticardiolipin, homocysteine, and factor V Leiden, was negative. Antithrombin III antigen was 68%, which was slightly low; however, the patient was on heparin therapy at the time these labs were drawn. Due to the extensive DVT, she underwent a venogram which showed stenosis of the left common iliac vein with well-established collaterals that led to the diagnosis of MTS (Figure 1 and Figure 2).

**Figure 3:**
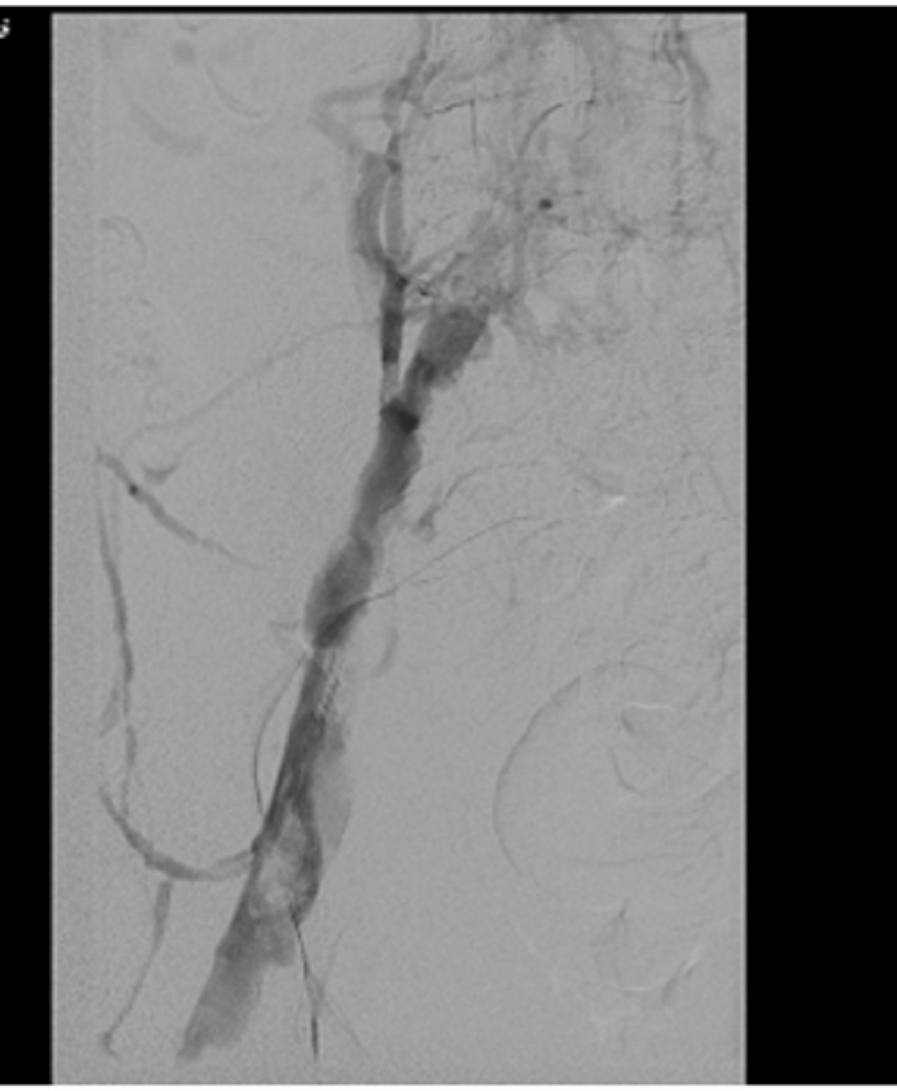
Improved flow after stent deployment.

She underwent angioplasty of the iliac vein using a 10 mm balloon that did not improve the flow, so a stent was deployed. A repeat venogram showed the vein was open with some residual thrombus more proximally (Figure 3).

Thrombolysis of the left lower extremity DVT and placement of an inferior vena cava (IVC) filter was also done at the same time, which she tolerated well. After the procedure, she was started on Coumadin® (warfarin) and argatroban was continued. She was later discharged home on Coumadin therapy.

## Discussion

MTS was first described by Virchow in 1851 when he noticed a left-sided predominance of iliofemoral DVT [4]. McMurich proposed the congenital nature of the syndrome in 1908 [5]. May and Thurner, in 1957, noticed that 22% of cadavers have the anatomic variant in which the right common iliac artery compresses the left iliac vein [1]. Cocket and Thomas carried out a clinical trial in 1965 by surgically exploring patients diagnosed with iliofemoral venous thrombosis, and they found fibrous obstruction of the left iliac vein in all of their patients [6]. The mechanism of thrombosis was described by May and Thurner as a chronic pulsatile compression of the left common iliac vein by the right common iliac artery causing the development of collagen scars or spurs from endothelium irritation [1]. This eventually leads to a partial or complete occlusion of the iliac vein, causing venous stasis. A recent study in Johns Hopkins was done to determine if the compression of the left common iliac vein by the right common iliac artery is associated with left-sided DVT. The authors reviewed cases of 230 patients who had a contrast CT scan of the pelvis before the diagnosis of unilateral DVT and concluded that >70% compression of the iliac vein is needed to cause DVT [7]. Most patients with MTS present with DVT (77%), followed by edema and pain without DVT (23%) [5]. Patients may not notice DVT as it sometimes resolves with time. Many cases are recurrent with a past workup negative for other etiologies of DVT or PE, and were previously discharged on anticoagulation therapy but present with the same complaint later [8]. Very few cases, however, present as PE rather than DVT. The gold standard technique for diagnosing MTS is the femoral stick venogram [9]. The area of compression or obstruction with documentation of collateral presence confirms the diagnosis [2]. A femoral stick venogram, although invasive, offers many advantages. This is the only technique that allows other procedures to be done at the same time such as thrombolysis, balloon angioplasty, stenting, and even placing an IVC filter [2]. Other methods of diagnosis include CT with contrast, intravascular ultrasound, and air plethysmography. A lower extremity venous duplex cannot diagnose MTS as it cannot assess vasculatures beyond the femoral veins. Guidelines for the treatment of MTS are not available in the literature due to the low incidence of the syndrome. However, it is understood that anticoagulation therapy alone is not enough to prevent a recurrence [9]. Early therapies include open repair with venous bypass and repositioning of the iliac artery. The results of these therapies were variable. The endovascular approach is becoming more prevalent in the treatment of MTS. Short-term studies and reviews recommend that a stent be placed to relieve the underlying compression of the venous system and to continue anticoagulation for 6 to 12 months postoperatively [8]. Endoluminal stenting is associated with low morbidity and high patency rates [10]. Long term studies are not presently available.

## Conclusions

MTS is still vastly under-recognized. It is important to include MTS as one of the mainstream differential diagnoses of DVT along with PE in young women, especially if the etiology is not obvious. An aggressive evaluation of PE and lower extremity DVT for MTS could decrease mortality and morbidity significantly. Prognosis with MTS is very good if promptly identified and treated with an endovascular approach.
